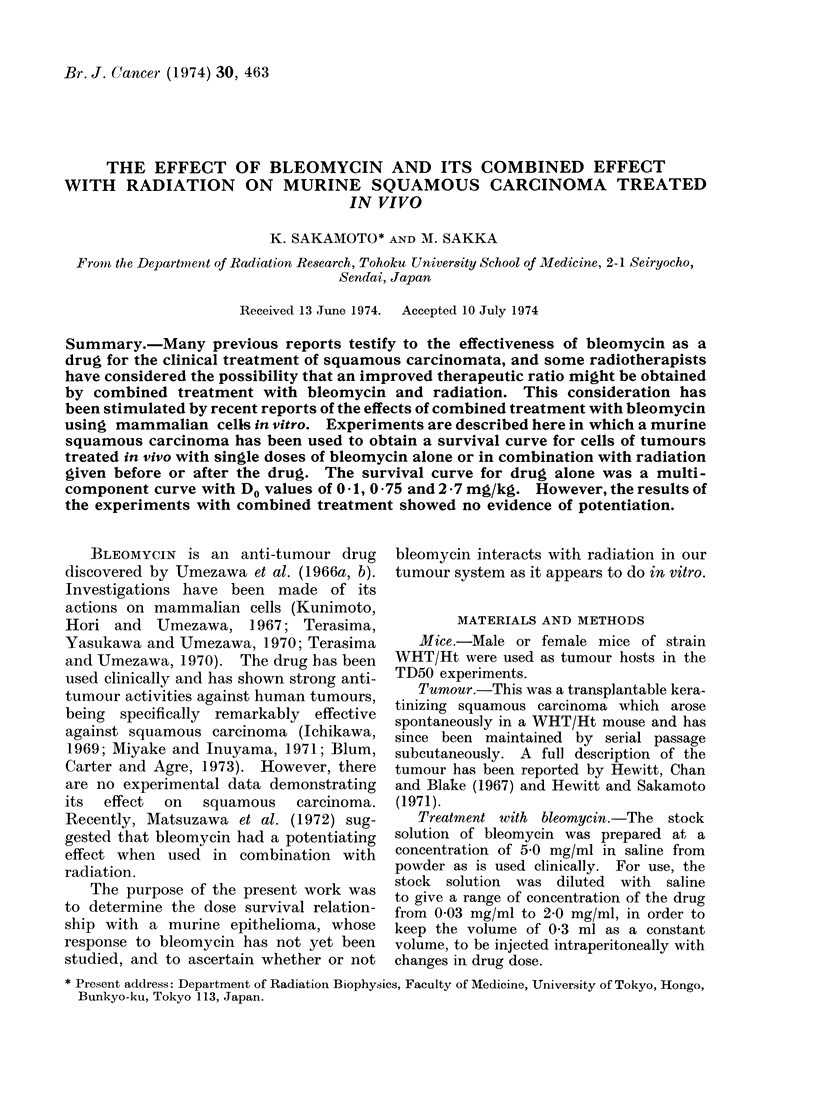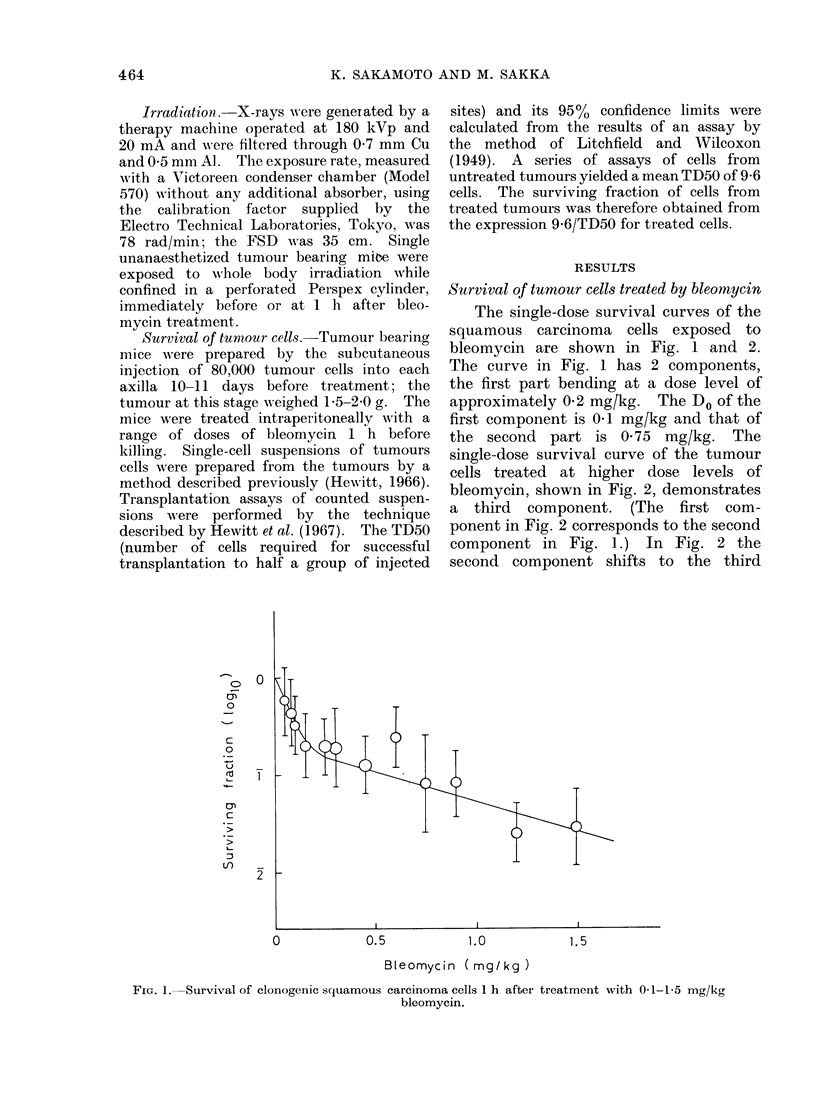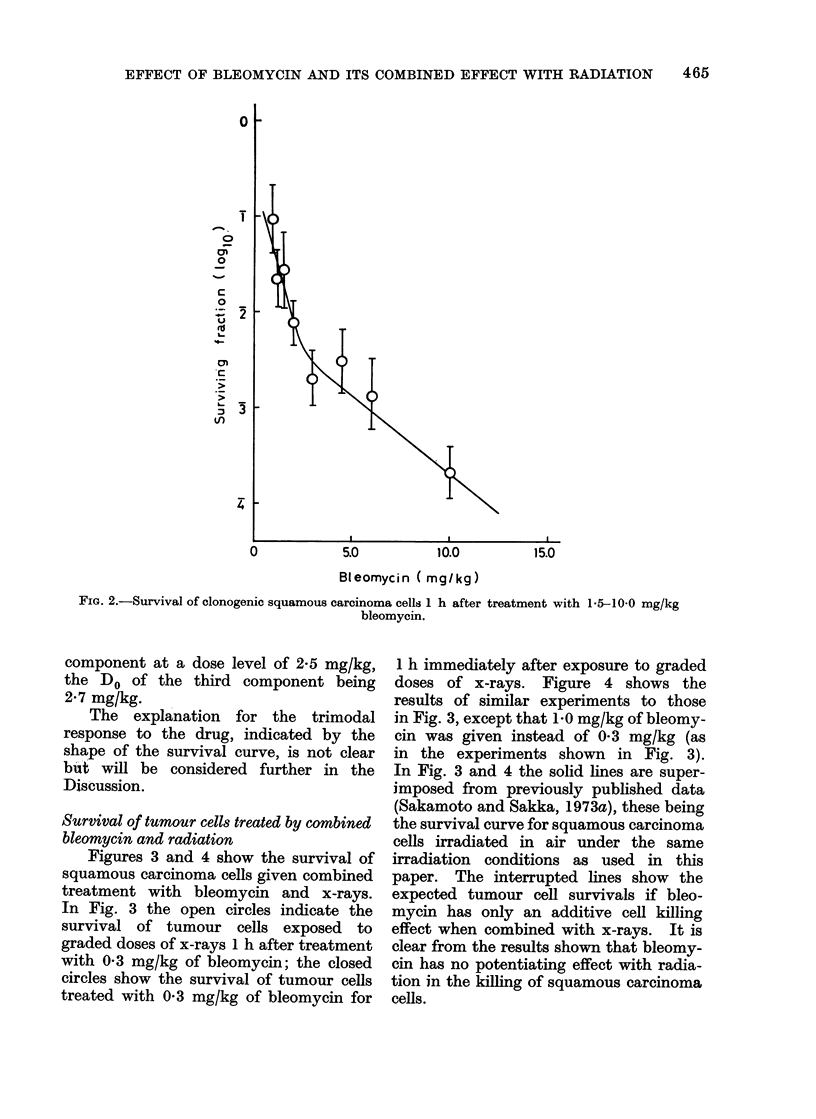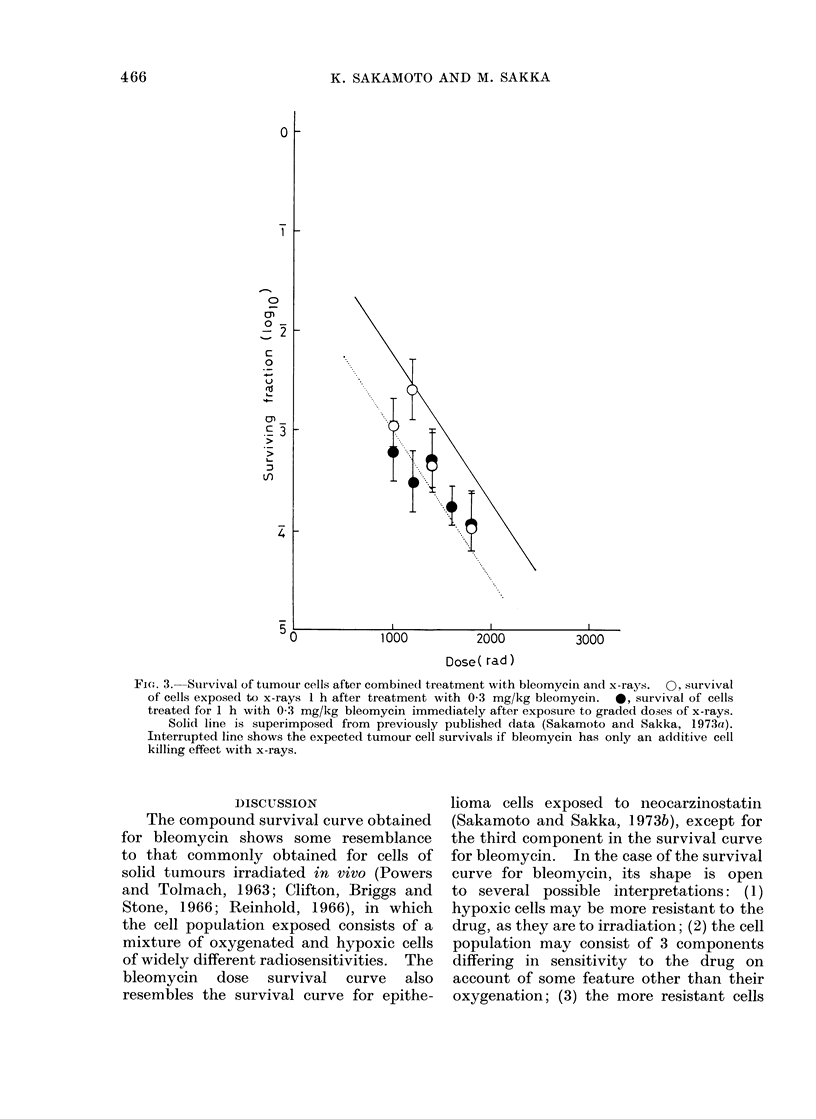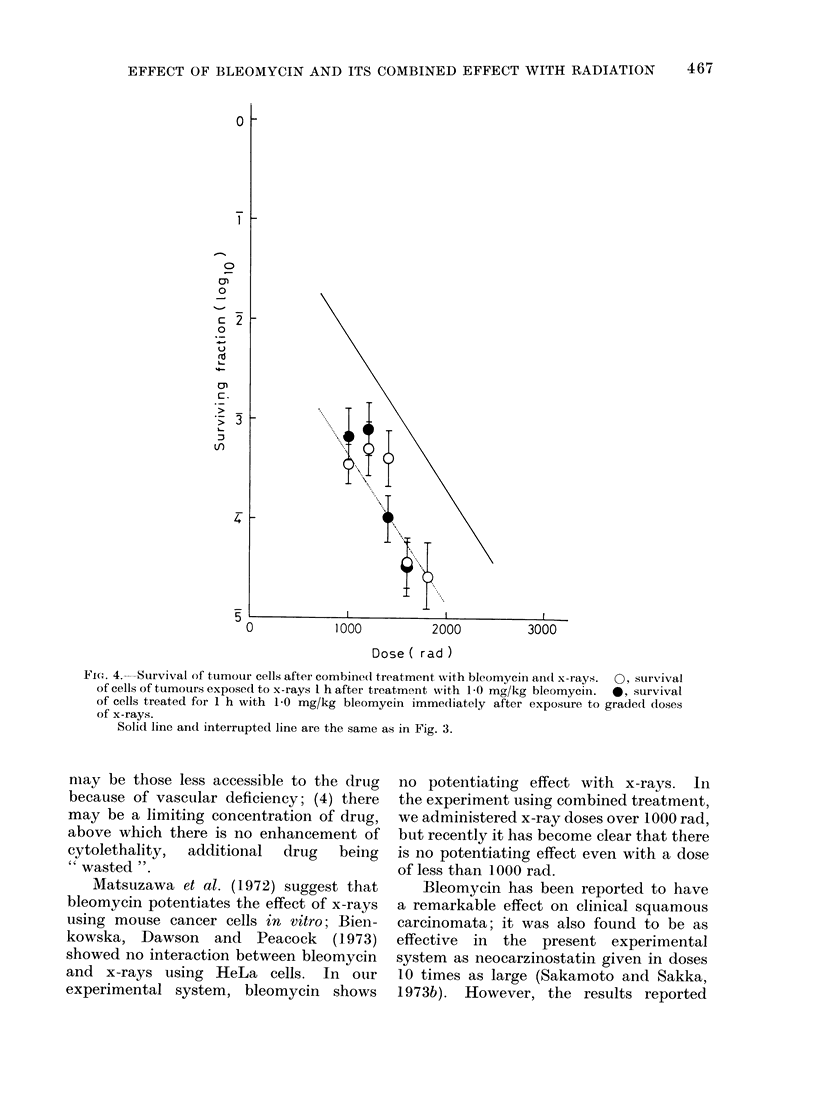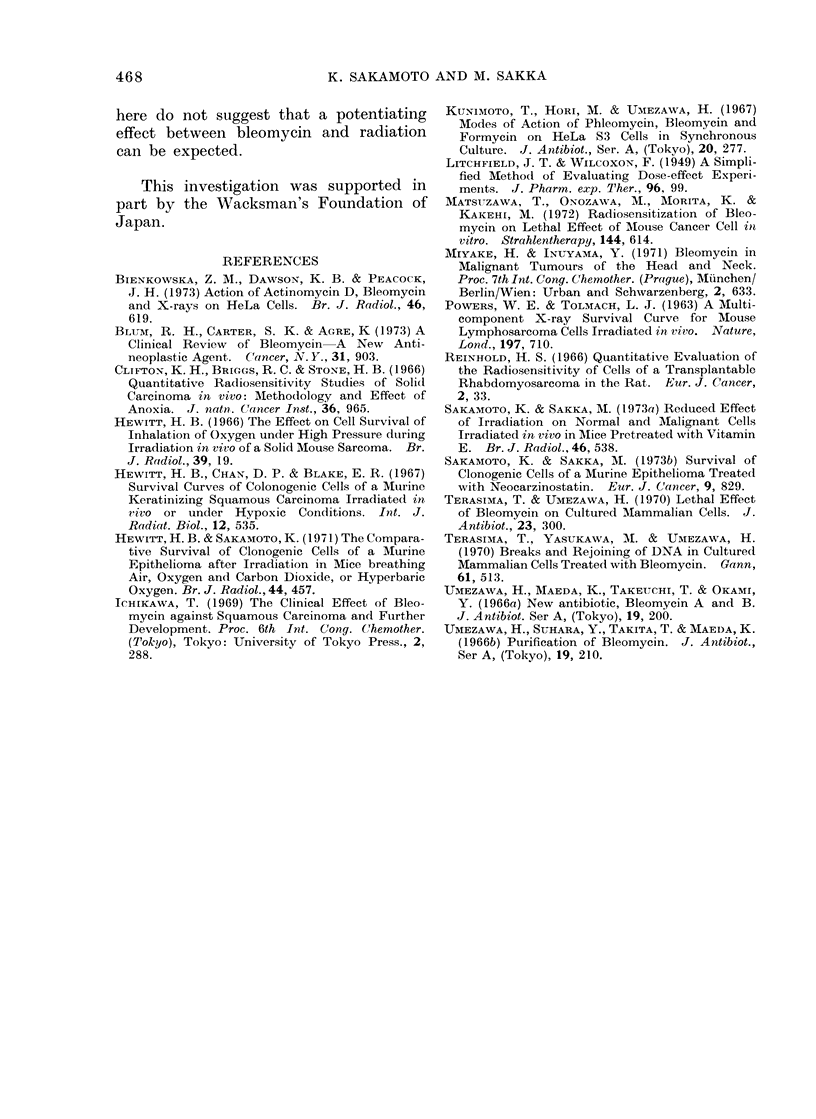# The Effect of Bleomycin and its Combined Effect with Radiation on Murine Squamous Carcinoma Treated in vivo

**DOI:** 10.1038/bjc.1974.221

**Published:** 1974-11

**Authors:** K. Sakamoto, M. Sakka

## Abstract

Many previous reports testify to the effectiveness of bleomycin as a drug for the clinical treatment of squamous carcinomata, and some radiotherapists have considered the possibility that an improved therapeutic ratio might be obtained by combined treatment with bleomycin and radiation. This consideration has been stimulated by recent reports of the effects of combined treatment with bleomycin using mammalian cells *in vitro.* Experiments are described here in which a murine squamous carcinoma has been used to obtain a survival curve for cells of tumours treated *in vivo* with single doses of bleomycin alone or in combination with radiation given before or after the drug. The survival curve for drug alone was a multicomponent curve with D_0_ values of 0·1, 0·75 and 2·7 mg/kg. However, the results of the experiments with combined treatment showed no evidence of potentiation.


*Br. J. Cancer* (1974) **30**, 463

### THE EFFECT OF BLEOMYCIN AND ITS COMBINED EFFECT WITH RADIATION ON MURINE SQUAMOUS CARCINOMA TREATED *IN VIVO*

K. SAKAMOTO* AND M. SAKKA

*From the Department of Radiation Research, Tohoku University School of Medicine, 2-1 Seiryocho, Sendai, Japan*

Received 13 June 1974. Accepted 10 July 1974

**Summary.—Many previous reports testify to the effectiveness of bleomycin as a drug for the clinical treatment of squamous carcinomata, and some radiotherapists have considered the possibility that an improved therapeutic ratio might be obtained by combined treatment with bleomycin and radiation. This consideration has been stimulated by recent reports of the effects of combined treatment with bleomycin using mammalian cells *in vitro*. Experiments are described here in which a murine squamous carcinoma has been used to obtain a survival curve for cells of tumours treated *in vivo* with single doses of bleomycin alone or in combination with radiation given before or after the drug. The survival curve for drug alone was a multi-component curve with $D_0$ values of 0·1, 0·75 and 2·7 mg/kg. However, the results of the experiments with combined treatment showed no evidence of potentiation.**

BLEOMYCIN is an anti-tumour drug discovered by Umezawa *et al.* (1966*a*, *b*). Investigations have been made of its actions on mammalian cells (Kunimoto, Hori and Umezawa, 1967; Terasima, Yasukawa and Umezawa, 1970; Terasima and Umezawa, 1970). The drug has been used clinically and has shown strong anti-tumour activities against human tumours, being specifically remarkably effective against squamous carcinoma (Ichikawa, 1969; Miyake and Inuyama, 1971; Blum, Carter and Agre, 1973). However, there are no experimental data demonstrating its effect on squamous carcinoma. Recently, Matsuzawa *et al.* (1972) suggested that bleomycin had a potentiating effect when used in combination with radiation.

The purpose of the present work was to determine the dose survival relationship with a murine epithelioma, whose response to bleomycin has not yet been studied, and to ascertain whether or not bleomycin interacts with radiation in our tumour system as it appears to do *in vitro*.

### MATERIALS AND METHODS

*Mice.*—Male or female mice of strain WHT/Ht were used as tumour hosts in the TD50 experiments.

*Tumour.*—This was a transplantable keratinizing squamous carcinoma which arose spontaneously in a WHT/Ht mouse and has since been maintained by serial passage subcutaneously. A full description of the tumour has been reported by Hewitt, Chan and Blake (1967) and Hewitt and Sakamoto (1971).

*Treatment with bleomycin.*—The stock solution of bleomycin was prepared at a concentration of 5·0 mg/ml in saline from powder as is used clinically. For use, the stock solution was diluted with saline to give a range of concentration of the drug from 0·03 mg/ml to 2·0 mg/ml, in order to keep the volume of 0·3 ml as a constant volume, to be injected intraperitoneally with changes in drug dose.

* Present address: Department of Radiation Biophysics, Faculty of Medicine, University of Tokyo, Hongo, Bunkyo-ku, Tokyo 113, Japan.

*Irradiation.*—X-rays were generated by a therapy machine operated at 180 kVp and 20 mA and were filtered through 0·7 mm Cu and 0·5 mm Al. The exposure rate, measured with a Victoreen condenser chamber (Model 570) without any additional absorber, using the calibration factor supplied by the Electro Technical Laboratories, Tokyo, was 78 rad/min; the FSD was 35 cm. Single unanaesthetized tumour bearing mice were exposed to whole body irradiation while confined in a perforated Perspex cylinder, immediately before or at 1 h after bleomycin treatment.

*Survival of tumour cells.*—Tumour bearing mice were prepared by the subcutaneous injection of 80,000 tumour cells into each axilla 10–11 days before treatment; the tumour at this stage weighed 1·5–2·0 g. The mice were treated intraperitoneally with a range of doses of bleomycin 1 h before killing. Single-cell suspensions of tumours cells were prepared from the tumours by a method described previously (Hewitt, 1966). Transplantation assays of counted suspensions were performed by the technique described by Hewitt *et al.* (1967). The TD50 (number of cells required for successful transplantation to half a group of injected sites) and its 95% confidence limits were calculated from the results of an assay by the method of Litchfield and Wilcoxon (1949). A series of assays of cells from untreated tumours yielded a mean TD50 of 9·6 cells. The surviving fraction of cells from treated tumours was therefore obtained from the expression 9·6/TD50 for treated cells.

### RESULTS

*Survival of tumour cells treated by bleomycin*

The single-dose survival curves of the squamous carcinoma cells exposed to bleomycin are shown in Fig. 1 and 2. The curve in Fig. 1 has 2 components, the first part bending at a dose level of approximately 0·2 mg/kg. The $D_0$ of the first component is 0·1 mg/kg and that of the second part is 0·75 mg/kg. The single-dose survival curve of the tumour cells treated at higher dose levels of bleomycin, shown in Fig. 2, demonstrates a third component. (The first component in Fig. 2 corresponds to the second component in Fig. 1.) In Fig. 2 the second component shifts to the third

FIG. 1.—Survival of clonogenic squamous carcinoma cells 1 h after treatment with 0·1–1·5 mg/kg bleomycin.

FIG. 2.—Survival of clonogenic squamous carcinoma cells 1 h after treatment with 1·5–10·0 mg/kg bleomycin.

component at a dose level of 2·5 mg/kg, the $D_0$ of the third component being 2·7 mg/kg.

The explanation for the trimodal response to the drug, indicated by the shape of the survival curve, is not clear but will be considered further in the Discussion.

*Survival of tumour cells treated by combined bleomycin and radiation*

Figures 3 and 4 show the survival of squamous carcinoma cells given combined treatment with bleomycin and x-rays. In Fig. 3 the open circles indicate the survival of tumour cells exposed to graded doses of x-rays 1 h after treatment with 0·3 mg/kg of bleomycin; the closed circles show the survival of tumour cells treated with 0·3 mg/kg of bleomycin for 1 h immediately after exposure to graded doses of x-rays. Figure 4 shows the results of similar experiments to those in Fig. 3, except that 1·0 mg/kg of bleomycin was given instead of 0·3 mg/kg (as in the experiments shown in Fig. 3). In Fig. 3 and 4 the solid lines are superimposed from previously published data (Sakamoto and Sakka, 1973*a*), these being the survival curve for squamous carcinoma cells irradiated in air under the same irradiation conditions as used in this paper. The interrupted lines show the expected tumour cell survivals if bleomycin has only an additive cell killing effect when combined with x-rays. It is clear from the results shown that bleomycin has no potentiating effect with radiation in the killing of squamous carcinoma cells.

FIG. 3.—Survival of tumour cells after combined treatment with bleomycin and x-rays. ○, survival of cells exposed to x-rays 1 h after treatment with 0·3 mg/kg bleomycin. ●, survival of cells treated for 1 h with 0·3 mg/kg bleomycin immediately after exposure to graded doses of x-rays. Solid line is superimposed from previously published data (Sakamoto and Sakka, 1973*a*). Interrupted line shows the expected tumour cell survivals if bleomycin has only an additive cell killing effect with x-rays.

### DISCUSSION

The compound survival curve obtained for bleomycin shows some resemblance to that commonly obtained for cells of solid tumours irradiated *in vivo* (Powers and Tolmach, 1963; Clifton, Briggs and Stone, 1966; Reinhold, 1966), in which the cell population exposed consists of a mixture of oxygenated and hypoxic cells of widely different radiosensitivities. The bleomycin dose survival curve also resembles the survival curve for epithelioma cells exposed to neocarzinostatin (Sakamoto and Sakka, 1973*b*), except for the third component in the survival curve for bleomycin. In the case of the survival curve for bleomycin, its shape is open to several possible interpretations: (1) hypoxic cells may be more resistant to the drug, as they are to irradiation; (2) the cell population may consist of 3 components differing in sensitivity to the drug on account of some feature other than their oxygenation; (3) the more resistant cells

FIG. 4.—Survival of tumour cells after combined treatment with bleomycin and x-rays. ○, survival of cells of tumours exposed to x-rays 1 h after treatment with 1·0 mg/kg bleomycin. ●, survival of cells treated for 1 h with 1·0 mg/kg bleomycin immediately after exposure to graded doses of x-rays.

Solid line and interrupted line are the same as in Fig. 3.

may be those less accessible to the drug because of vascular deficiency; (4) there may be a limiting concentration of drug, above which there is no enhancement of cytolethality, additional drug being " wasted ".

Matsuzawa *et al.* (1972) suggest that bleomycin potentiates the effect of x-rays using mouse cancer cells *in vitro*; Bienkowska, Dawson and Peacock (1973) showed no interaction between bleomycin and x-rays using HeLa cells. In our experimental system, bleomycin shows no potentiating effect with x-rays. In the experiment using combined treatment, we administered x-ray doses over 1000 rad, but recently it has become clear that there is no potentiating effect even with a dose of less than 1000 rad.

Bleomycin has been reported to have a remarkable effect on clinical squamous carcinomata; it was also found to be as effective in the present experimental system as neocarzinostatin given in doses 10 times as large (Sakamoto and Sakka, 1973*b*). However, the results reported here do not suggest that a potentiating effect between bleomycin and radiation can be expected.

This investigation was supported in part by the Wacksman's Foundation of Japan.

### REFERENCES

BIENKOWSKA, Z. M., DAWSON, K. B. & PEACOCK, J. H. (1973) Action of Actinomycin D, Bleomycin and X-rays on HeLa Cells. *Br. J. Radiol.*, **46**, 619.

BLUM, R. H., CARTER, S. K. & AGRE, K (1973) A Clinical Review of Bleomycin—A New Antineoplastic Agent. *Cancer, N.Y.*, **31**, 903.

CLIFTON, K. H., BRIGGS, R. C. & STONE, H. B. (1966) Quantitative Radiosensitivity Studies of Solid Carcinoma *in vivo*: Methodology and Effect of Anoxia. *J. natn. Cancer Inst.*, **36**, 965.

HEWITT, H. B. (1966) The Effect on Cell Survival of Inhalation of Oxygen under High Pressure during Irradiation *in vivo* of a Solid Mouse Sarcoma. *Br. J. Radiol.*, **39**, 19.

HEWITT, H. B., CHAN, D. P. & BLAKE, E. R. (1967) Survival Curves of Colonogenic Cells of a Murine Keratinizing Squamous Carcinoma Irradiated *in vivo* or under Hypoxic Conditions. *Int. J. Radiat. Biol.*, **12**, 535.

HEWITT, H. B. & SAKAMOTO, K. (1971) The Comparative Survival of Clonogenic Cells of a Murine Epithelioma after Irradiation in Mice breathing Air, Oxygen and Carbon Dioxide, or Hyperbaric Oxygen. *Br. J. Radiol.*, **44**, 457.

ICHIKAWA, T. (1969) The Clinical Effect of Bleomycin against Squamous Carcinoma and Further Development. *Proc. 6th Int. Cong. Chemother. (Tokyo)*, Tokyo: University of Tokyo Press., **2**, 288.

KUNIMOTO, T., HORI, M. & UMEZAWA, H. (1967) Modes of Action of Phleomycin, Bleomycin and Formycin on HeLa S3 Cells in Synchronous Culture. *J. Antibiot.*, Ser. A, (Tokyo), **20**, 277.

LITCHFIELD, J. T. & WILCOXON, F. (1949) A Simplified Method of Evaluating Dose-effect Experiments. *J. Pharm. exp. Ther.*, **96**, 99.

MATSUZAWA, T., ONOZAWA, M., MORITA, K. & KAKEHI, M. (1972) Radiosensitization of Bleomycin on Lethal Effect of Mouse Cancer Cell *in vitro*. *Strahlentherapy*, **144**, 614.

MIYAKE, H. & INUYAMA, Y. (1971) Bleomycin in Malignant Tumours of the Head and Neck. *Proc. 7th Int. Cong. Chemother. (Prague)*, München/Berlin/Wien: Urban and Schwarzenberg, **2**, 633.

POWERS, W. E. & TOLMACH, L. J. (1963) A Multicomponent X-ray Survival Curve for Mouse Lymphosarcoma Cells Irradiated *in vivo*. *Nature, Lond.*, **197**, 710.

REINHOLD, H. S. (1966) Quantitative Evaluation of the Radiosensitivity of Cells of a Transplantable Rhabdomyosarcoma in the Rat. *Eur. J. Cancer*, **2**, 33.

SAKAMOTO, K. & SAKKA, M. (1973*a*) Reduced Effect of Irradiation on Normal and Malignant Cells Irradiated *in vivo* in Mice Pretreated with Vitamin E. *Br. J. Radiol.*, **46**, 538.

SAKAMOTO, K. & SAKKA, M. (1973*b*) Survival of Clonogenic Cells of a Murine Epithelioma Treated with Neocarzinostatin. *Eur. J. Cancer*, **9**, 829.

TERASIMA, T. & UMEZAWA, H. (1970) Lethal Effect of Bleomycin on Cultured Mammalian Cells. *J. Antibiot.*, **23**, 300.

TERASIMA, T., YASUKAWA, M. & UMEZAWA, H. (1970) Breaks and Rejoining of DNA in Cultured Mammalian Cells Treated with Bleomycin. *Gann*, **61**, 513.

UMEZAWA, H., MAEDA, K., TAKEUCHI, T. & OKAMI, Y. (1966*a*) New antibiotic, Bleomycin A and B. *J. Antibiot.* Ser A, (Tokyo), **19**, 200.

UMEZAWA, H., SUHARA, Y., TAKITA, T. & MAEDA, K. (1966*b*) Purification of Bleomycin. *J. Antibiot.*, Ser A, (Tokyo), **19**, 210.